# Pesticides in Drinking Water – The Brazilian Monitoring Program

**DOI:** 10.3389/fpubh.2015.00246

**Published:** 2015-11-04

**Authors:** Auria M. C. Barbosa, Marize de L. M. Solano, Gisela de A. Umbuzeiro

**Affiliations:** ^1^Brazilian Health Surveillance Agency (ANVISA), Brasilia, Brazil; ^2^School of Technology, University of Campinas, Limeira, Brazil

**Keywords:** drinking water criteria, drinking water standards, pesticide risk, Sisagua, Vigiagua

## Abstract

Brazil is the world largest pesticide consumer; therefore, it is important to monitor the levels of these chemicals in the water used by population. The Ministry of Health coordinates the National Drinking Water Quality Surveillance Program (Vigiagua) with the objective to monitor water quality. Water quality data are introduced in the program by state and municipal health secretariats using a database called Sisagua (Information System of Water Quality Monitoring). Brazilian drinking water norm (Ordinance 2914/2011 from Ministry of Health) includes 27 pesticide active ingredients that need to be monitored every 6 months. This number represents <10% of current active ingredients approved for use in the country. In this work, we analyzed data compiled in Sisagua database in a qualitative and quantitative way. From 2007 to 2010, approximately 169,000 pesticide analytical results were prepared and evaluated, although approximately 980,000 would be expected if all municipalities registered their analyses. This shows that only 9–17% of municipalities registered their data in Sisagua. In this dataset, we observed non-compliance with the minimum sampling number required by the norm, lack of information about detection and quantification limits, insufficient standardization in expression of results, and several inconsistencies, leading to low credibility of pesticide data provided by the system. Therefore, it is not possible to evaluate exposure of total Brazilian population to pesticides via drinking water using the current national database system Sisagua. Lessons learned from this study could provide insights into the monitoring and reporting of pesticide residues in drinking water worldwide.

## Introduction

In 1997, at Mar del Plata, the Action Plan from the United Nations Water Conference recognized water as a right for the first time and, in 2010, the same organization stated that a sufficient and safe supply of water is essential for the realization of many other human rights (1). Since the 70s, the global population has nearly doubled, while the urban population almost tripled, in similar amount as the number of people using drinking water sources (2, 3). To serve public health, economic and human rights necessities, monitoring programs are used to track global, regional, and national progress on access to drinking water and sanitation (4). The lack of data regarding the occurrence of contaminants in waters inhibits the prioritization of substances to be regulated and the establishment of criteria for drinking water in relation to the risks associated with drinking water consumption (5). The selection of compounds to be regulated is not easy and quantity, physical and chemical properties, occurrence and potential hazard to non-target species need to be considered, for example (6).

Generally, when pesticide is applied following Good Agricultural Practices (GAP), the acceptable Maximum Residue Limits (MRLs) are not exceeded (7, 8). When a pesticide is approved, these maximum residue levels must not present risk to human health. However, the misuse of pesticides can occur and concentrations above the MRL can be found in crops (7, 8). Pesticide residues also can reach surface and groundwater, and consequently expose humans via drinking water. The contamination of water bodies can occur by leaching processes from plants and soil followed by rainwater drainage in rural and urban environments, as well as from sewage discharges, because of pesticides uses in pets and gardens.

To establish drinking water standards for chemical substances, a Chemical Risk Quantitative Assessment methodology has been used. The steps are hazard identification, exposure assessment, dose-response evaluation, and risk characterization (9, 10). The World Health Organization (WHO) and the Organization for Food and Agriculture of the United Nations (FAO) have established acceptable daily intake levels (ADIs) of pesticide residues. ADIs are values that indicate the maximum daily intake of a substance that does not represent risk to human health throughout the individual’s life. Therefore, a pesticide ADI is usually obtained from its NOEL or NOAEL (no-observed-effect-level, or no-observed-adverse-effect-level), estimated from toxicity studies with laboratory animals with appropriate safety factors (varying from 10 to 10,000) (11, 12). However, the additional risk due to simultaneous exposure to several substances and different forms of exposure (i.e., drinking water, plant and animal foods consumption, dermal exposure, etc.) cannot be disregarded because synergism can occur (13, 14).

Many factors are involved in the establishment of a drinking water standard. Neto made a comparison in 2010 between the patterns of Brazilian drinking water criteria, international guidelines, and other countries data, finding a great variability in the way of establishing these values (15). The United States, for example, when establishing their criteria, take into account the potential adverse effects of contaminants on human health, the frequency and level of occurrence in public water supply systems, the available treatment technologies, and if the cost of regulation of the substance will represent a significant opportunity to reduce risks to public health (16). Otherwise, the values defined in Directive 98/83/EC adopted by the Member States of the European Community are not based on the chemical’s toxicological properties, differently from WHO guidelines and those of other countries, but in the assumption that these substances must not be present in the drinking water, using a pragmatic cut off value of 0.1 μg/L for single pesticide and 0.5 μg/L for the sum of those present (17). Australia has a default value for pesticides, which is the quantification limit of the analytical method, therefore the quality criteria is not based on the toxicological properties of the substances, unless the analytical quantification limit is too high (18, 19). Therefore, setting a drinking water standard is not an easy task and includes not only scientifically but also economic, technological, and political factors.

With regard to the approach used by the United States, there are water monitoring programs to verify the occurrence of regulated and non-regulated compounds. This information is used to help in the definition of new priority contaminants that will be listed in the Drinking Water Contaminant Candidate List. This list will be used by the US EPA to define the need of the inclusion of new compounds in the drinking water standard. Their regulatory infrastructure is based on good practice analytical methods, laboratory certification, treatment technology (to identify and/or develop high quality, cost-effective treatment technologies to meet regulation), a periodical review of standards, the National Contaminant Occurrence Database, and the non-regulated contaminant candidates. This list is divided as follows: substances that are priorities for additional research, those that need additional occurrence data, and those that are priorities for consideration in rulemaking (20). This Non-regulated Contaminants Monitoring Program could guide developing countries such as Brazil for the inclusion of priority compounds in a drinking water norm.

Since 2008, Brazil is leading the global consumption of agrochemicals, a position previously occupied by the United States (1, 2). In addition to protecting crops from pests, diseases, and weeds, pesticides also pose a risk to human health and the environment through contamination of food, agricultural soil, surface, and ground water. Brazilian consumption of pesticides reached around 496,000 tons of active ingredients in 2013 according to the last report available (1, 2). Suitable chemical analytical methods are needed for the detection of pesticides and emerging contaminants. Recently, a method for quantifying several pesticide residues in water was developed and used to test drinking water samples from 9 cities, and surface waters from 13 rivers of the State of São Paulo, Brazil after 1 year of sampling collection (21). This was not the first time that difenoconazol, epoxiconazole, tebuconazole, atrazine, azoxystrobin, carbendazim, and fipronil were detected in Brazilian water bodies (21–24). One of the rivers that is the main source of drinking water to the city of Campinas have been studied for several years for the presence of emerging contaminants (5, 6, 21, 23) and endrocrine-active compounds (25). Recently, an *in vivo* study conducted with drinking water samples from this river showed evidence of endocrine disruption in prepubertal female rats (26).

Currently, in Brazil, there are 380 active ingredients authorized by the Ministry of Agriculture for pesticides used on crops and 1,670 formulated plant protection products on the market (27). Pesticide registration is regulated by Decree No. 4074/2002. It is a shared responsibility of the Ministry of Agriculture, Livestock and Supply (MAPA), Ministry of the Environment (MMA), and Ministry of Health (MH). The Ministry of Health is responsible for the analysis of the health aspects of the registration procedure and also for monitoring pesticides in food (among other activities). One of its departments, the Brazilian Health Surveillance Agency (ANVISA) coordinates the Pesticide Residues Analysis Program in Food (PARA). For example, in 2010, 28% of the samples were found unsatisfactory because of the presence of unauthorized pesticide residues or authorized ones above the MRLs (28).

Drinking water quality is not regulated by ANVISA but by the General Coordination of Health Surveillance (CGVAM) from Health Surveillance Secretariat (SVS), also sectors of the MH. The drinking water norm that is in place is the Ordinance No. 2914/11 and it defines standards and procedures related to the control and surveillance of water quality. CGVAM also coordinates the National Monitoring Water Quality for Human Consumption Program (Vigiagua), a monitoring water quality program that operates through the Monitoring Information on Water Quality for Human Consumption System (Sisagua). Sisagua compiles the data that is included in the database. The drinking water suppliers are responsible for the quality control of drinking water; however, the water quality surveillance activity is a task of CGVAM, in collaboration with state and municipal secretariats (7).The latter are responsible for the inclusion of the data in the Sisagua database. In summary, the norm indicates that the data on the drinking water quality needs to be provided to MH through Sisagua, and then, the public health authorities are able to verify if the water consumed by the population complies with the current regulation, including with regard to the risks it may pose to human health.

The water quality Ordinance MH No. 2914/2011 regulates 64 chemical substances, of which 27 are pesticides monitored every six months and with data insertion in Sisagua. Table 1 shows the regulated pesticides and their Maximum Allowed Concentrations (MAC).

**Table 1:** **Pesticides regulated by Brazilian Ordinance MH No. 2914/2011 and their maximum allowed concentrations (MAC) (29)**.

Pesticide (active ingredient)	CAS registry number	MAC (**μ**g/L)	Pesticide (active ingredient)	CAS registry number	MAC (**μ**g/L)
2,4-D + 2,4,5 T	94-75-7	30	Lindane (γ HCH)	58-89-9	2
	93-76-5				
Alachlor	15972-60-8	20	Mancozeb	8018-01-7	180
Aldicarb + aldicarbsulfone + aldicarbsulfoxide	116-06-3	10	Methamidophos	10265-92-6	12
	1646-88-4				
	1646-87-3				
Aldrin + dieldrin	309-00-2	0.03	Metolachlor	51218-45-2	10
	60-57-1				
Atrazine	1912-24-9	2	Molinate	2212-67-1	6
Carbendazim + benomil	10605-21-7	120	Parathion-methyl	298-00-0	9
	17804-35-2				
Carbofuran	1563-66-2	7	Pendimethalin	40487-42-1	20
Chlordane	5103-74-2	0.2	Permethrin	52645-53-1	20
Chlorpyrifos + chlorpyrifos − oxon	2921-88-2	30	Profenophos	41198-08-7	60
	5598-15-2				
DDT + DDD + DDE	50-29-3	1	Simazine	122-34-9	2
	72-54-8				
	72-55-9				
Diuron	330-54-1	90	Tebuconazole	107534-96-3	180
Endosulfan (α, β, and salt)	115-29-7	20	Terbuphos	13071-79-9	1.2
	959-98-8				
	33213-65-9				
	1031-07-8				
Endrin	72-20-8	0.6	Trifluralin	1582-09-8	20
Glyphosate + AMPA	1071-83-6	500			
	1066-51-9				

The aim of this study was to evaluate the monitoring of pesticides data from the National Monitoring Water Quality for Human Consumption Program (Vigiagua), available on the Monitoring Information on Water Quality for Human Consumption System (Sisagua). Therefore, in this paper, we will critically evaluate the inclusion, compilation process, and assessment of pesticides data in the drinking water database available from the Vigiagua federal program.

## Materials and Methods

### Water Quality Control

Quality control of drinking water in Brazil is assured through the evaluation of several parameters, which include microbiological, physical–chemical, and pesticides analyses (for details, please see Ordinance MH No. 2914/2011). The laboratories must perform their analyses under quality control systems, e.g., ISO17025 (30). Unfortunately, no information on the analytical methods applied was available in the Sisagua dataset.

### Vigiagua Pesticides Data Analyses

CGVAM/MH provided the monitoring data set corresponding to the years 2007–2010 because the Sisagua dataset is not publicly available. The Brazilian drinking water ordinance states that analysis of pesticides must be performed in the water produced by the Drinking Water Treatment Plant (DWTP). If a sample presents a result not in compliance with the norm, the same pesticides should be then analyzed in the respective distribution network. As a consequence, limited data on the distribution network were retrieved, and therefore, only data from DWTPs were considered in our analyses.

We excluded invalid results in our data analysis after we observed different types of inconsistencies in the data set and reported them in number of non-valid results. Pesticide active ingredients in drinking water were reported by region, state, state capitals, and other municipalities (31). The verification of compliance with the drinking water standard was performed using the previous Ordinance MH No. 518/04, because during the period of this research it was the norm in place. When the information was reported as below certain value, we assumed that this was the limit of quantification and, if this was above the maximum allowed concentrations, the sample was considered non-compliant with the norm.

### Evaluation of Pesticides Under the Current Ordinance MH No. 2914/2011

A survey was conducted on the best-selling active ingredients in Brazil to assess whether the regulated pesticides in the current drinking water were representative. The survey was based on the marketing data from ANVISA (from 2nd half of 2010 and 1st half of 2011), the Agrofit system (System of Phytosanitary Pesticides from the MAPA) and the most recent Pesticides Trading Report, released by IBAMA (Brazilian Institute of Environment and Renewable Natural Resources) (27, 32, 33). We considered only the most sold pesticide active ingredients in Brazil, from 2009 to 2012, which were used in a minimum of 1,000 tons/year. This list was compared with the Ordinance MH 2914/11, as well as with the canceled pesticides or the ones in registration revaluation (27, 33, 34). For information we consulted the monographs or toxicological reassessment files available at the official website of ANVISA. The information about the registered pesticides in Brazil was obtained in Agrofit (27, 32, 34).

### Drinking Water Quality Criteria Calculation

Drinking water criteria were calculated using the ADIs publicly available in the ANVISA monographs, and the proposed WHO algorithm, applying 20% of allocation factor, 60 kg of body weight and 2 L of water consumption per person per day (10, 32, 35, 36).

## Results

### Pesticide Active Ingredients Consumed in Brazil

The pesticide active ingredients most consumed in Brazil from 2009 to 2012 were glyphosate, mineral oil, 2,4-D, atrazine, sulfur, methamidophos, vegetable oil, carbendazim, acephate, mancozeb, and diuron. Table 2 shows data on the substances whose sales were more than 1,000 tons in each reporting year, accounting for more than 80% of total sales (33).

**Table 2:** **The highest volume pesticide active ingredients in Brazil from 2009 to 2012 (above 1,000 tons/year)**.

Pesticide (active ingredient)	2009	2010	2011	2012
**2,4-D**	12,116.12	19,450.29	23,116.97	32,163.99
**Acephate**	5,204.89	5,233.44	8,124.83	13,080.63
Ametryn	1,624.09	2,858.40	3,441.88	4,705.76
**Atrazine**	10,133.80	12,811.48	18,580.93	27,139.56
Azoxystrobin	–	–	–	1,634.41
Bentazone	1,017.28	1,064.48	–	–
**Carbendazim**	6,712.59	7,629.82	12,216.92	7,999.80
Carbofuran	–	2,178.80	–	–
Chlorothalonil	1,964.75	2,488.77	3,001.41	2,987.65
**Chlorpyrifos**	2,966.39	3,191.78	4,288.36	6,218.35
Cipermetrine	–	–	3,219.22	–
Ciproconazol	–	1,707.27	1,653.27	1,090.87
Clomazone	2,712.01	5,255.42	6,171.87	4,731.45
Copper hydroxide	1,047.75	2,355.71	2,571.59	2,566.66
Copper oxychloride	3,152.99	3,364.24	3,706.01	3,854.88
Cymoxanil	1,189.55	–	–	–
**Diuron**	2,147.97	6,123.86	6,978.62	8,502.78
Endosulfan^a^	2,980.42	6,083.34	3,631.37	–
Etefom	–	–	1,244.48	1,554.26
Fipronil	–	–	–	1,068.60
Fluazinam	–		1,028.86	–
Flutriafol	–	–	–	1,044.19
**Glyphosate**	118,484.57	127,585.92	128,514.31	186,483.39
Glyphosate, isopropylamine salt	–	6,531.37	3,383.68	1,293.79
Hexazinone	–	1,155.16	1,560.75	2,009.96
**Imidacloprid**	1,399.15	2,441.11	5,074.00	5,476.11
Malathion	1,057.67	1,464.41	2,334.28	4,147.18
**Mancozeb**	3,590.35	6,917.62	7,290.18	7,134.82
Methamidophos^b^	10,774.80	17,661.77	12,838.84	–
**Methomyl**	–	3,350.53	4,247.09	6,376.02
Mineral oil	32,634.09	40,967.83	44,561.90	36,962.20
MSMA – monosodium methyl arsenate	1,399.88	1,672.78	1,515.11	1,778.80
**Paraquat dichloride**	1,977.19	3,113.24	4,275.38	5,249.54
Parathion-methyl	2,691.33	1,743.90	1,225.79	1,763.44
Picloram	–	–	1,485.90	1,625.86
Serricornim	–	–	–	3,612.38
Simazine	–	–	1,025.82	–
Sulfur	11,514.80	12,343.12	14,133.51	9,678.46
Tebuconazole	2,676.88	2,066.78	1,441.43	1,430.00
Tebuthiuron	–	2,041.97	3,195.36	3,650.86
Thiophanate methyl	3,754.32	4,472.94	4,947.79	4,800.58
Trifluralin	–	1,380.68	1,824.04	1,467.41
Vegetal oil	13,422.60	8,488.43	7,758.19	7,770.64
Total	260,348.23 (86.7%)^c^	327,196.66 (85.1%)^c^	355,609.94 (84.2%)^c^	413,055.28 (86.4%)^c^
Other active ingredients	40,001.47	57,304.62	66,632.32	64,737.16
Total of sales	300,349.70	384,501.28	422,242.26	477,792.44

*^a^Canceled by ANVISA in 2013*.

*^b^Canceled by ANVISA (Brazilian Health Surveillance Agency) in 2012*.

*^c^% related to the total of pesticides sold in the country. Pesticides with more than 5,000 ton sales in 2012 are highlighted in bold*.

### Vigiagua Data Analysis

#### Participation Assessment of Municipalities by State and Region

Geographically, the Brazilian states are grouped in regions for statistical interpretations, common public service management systems and implementation of public policies of the federal and state governments. Currently, there are five official regions: Midwest, Northeast, North, Southeast, and South. Area, population and Gross Domestic Product (GDP) are presented in Table 3. The North and Midwest regions have the largest areas, but the smallest population density and the lowest GDP, and it is where the federal district is located. The Northeast region has the third highest GDP; the Southeast has the highest GDP with the highest population density and it is where the two most populous cities are located: São Paulo, with 11 million inhabitants and Rio de Janeiro with 6 million. The South has the smallest area and a middle-size population, but is the second richest region in the country, and the one with the highest Human Development Index (HDI), the highest literacy rate and levels of education, health and social welfare of the country.

**Table 3:** **Geo-economic characteristics of Brazilian states by region**.

Region	Area (km^2^)	% of national territory	Population	% of population	GDP US$ thousands (2012)
North	3,869,638	45.2	17,231,027	8.50	115,691,500
Northeast	1,556,001	18.2	56,186,190	27.71	297,691,000
South	600,316	6.8	29,016,114	14.31	350,177,339
Southeast	927,286	10.9	85,115,623	41.9	1,194,091,133
Midwest	1,612,077	18.86	15,219,608	7.51	215,231,500

*Data from IBGE – (Brazilian Institute of Geography and Statistics) (2014) (37); GDP: Gross Domestic Product (estimated in US dollars)*.

The data available in Sisagua comes from the municipalities (state cities) of the Center-West, Southeast, and Southern regions of Brazil. The participation of municipalities in the North and Northeast was poor and did not contribute significantly to the data in the system. Table 4 shows the number of municipalities per state and region and the number of those that contributed pesticides data to Sisagua from 2007 to 2010 (31). We observed that the municipalities’ participation increased, although not consistently, along the years.

**Table 4:** **Number of Brazilian municipalities by state and region and the number that recorded data in Sisagua (2007–2010)**.

Region	State	Number of municipalities	Number of municipalities with results in Sisagua			
			2007	2008	2009	2010
Midwest	Distrito Federal	1	–	–	1	–
	Goiás	246	1	31	15	77
	Mato Grosso do Sul	78	8	26	24	29
	Mato Grosso	141	1	7	14	20
Subtotal		466	10	64	54	126
Northeast	Alagoas	102	–	–	–	–
	Bahia	417	–	24	15	6
	Ceará	184	–	47	4	1
	Maranhão	217	–	–	–	–
	Paraíba	223	–	–	–	–
	Pernambuco	185	–	–	–	1
	Piauí	224	–	1	–	–
	Rio Grande do Norte	167	1	–	–	1
	Sergipe	75	–	2	3	3
Subtotal		1,794	1	74	22	12
North	Acre	22	–	–	–	–
	Amazonas	62	–	1	–	–
	Amapá	16	–	–	–	–
	Pará	143	–	–	–	–
	Rondônia	52	–	–	–	–
	Roraima	15	–	–	–	–
	Tocantins	139	–	1	3	12
Subtotal		449	–	2	3	12
Southeast	Espírito Santo	78	2	6	5	4
	Minas Gerais	853	72	245	181	246
	Rio de Janeiro	92	–	7	4	9
	São Paulo	645	31	42	32	201
Subtotal		1,668	105	300	222	460
South	Paraná	399	347	352	270	252
	Rio Grande do Sul	496	61	83	42	4
	Santa Catarina	293	3	39	31	73
Subtotal		1,188	411	474	343	329
Total		5,565	527	914	644	939

#### Pesticides Data from Sisagua

**Figure 1:**
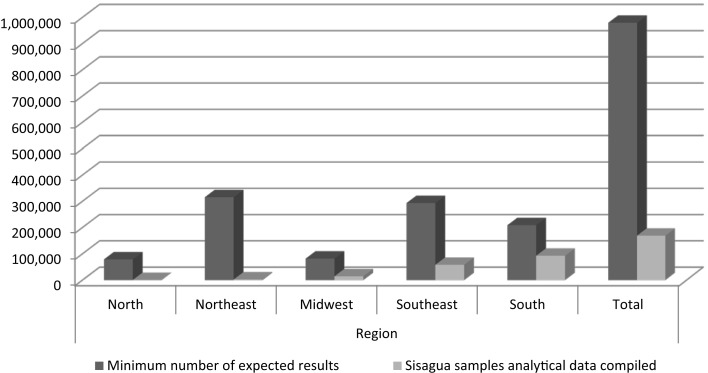
**The expected and actual number of pesticides analyses informed by each Brazilian region in Sisagua from 2007 to 2010**.

Taking into account, the Canceled number of municipalities that provided data in the system, failure to comply with the minimum Brazilian drinking water norm sampling request was also observed. Assuming that all municipalities have at least one DWTP and a minimum of two samples per year analyzed, we would expect at least 979,440 records in Sisagua during the studied period. However, only 169,080 (17%) were found. Failure to comply with the minimum pesticides analysis required by of the norm is therefore observed for all regions of Brazil (Figure 1).

#### Compliance to the Ordinance

The percentage of results above the drinking water standard ranged from 0.1 to 0.4%. Of the non-compliances (414), the highest percentage was for aldrin and dieldrin (38%), chlordane (19%), heptachlor and heptachlor epoxide (16%), endrin (7%), atrazine (5%), and other pesticides (15%). The non-compliance events could be related to the compounds with the lowest standard values, which suggest the need of a review in the analytical procedures to verify if false positives are being detected.

#### Sisagua Data Quality

To verify if a sample is in compliance with the drinking water quality standard, a suitable analytical method power (LOQ – Limit of Quantification) is necessary. Usually a “desirable LOQ” is 30% of the established standard (38, 39). The recorded data in Sisagua did not indicate the LOD and/or LOQ (Limit of Detection and/or Limit of Quantification) or the analytical methods used. We observed that 10–30% of the reported analyses were considered as not valid, mainly because of inconsistencies in the data, such as: (a) lack of information on the LOD and the LOQ of the analytical method used; (b) typing errors, the use of unidentified acronyms, numerically unacceptable expression of results, and no standardization on the number of decimal figures for the same analytical method measurement; (c) a high number of identical results, expressed in whole numbers, for different pesticide and for the same pesticide within the same drinking water provider; (d) results expressed as less than a value that was actually, above the standard established by the norm; and (e) several results reported as “not detected” preventing us from verifying compliance of the sample with the norm because of lack of information on the LOD/LOQ of the analytical method used. Table 5 summarizes the available data and the results considered as valid.

**Table 5:** **Number of pesticides analysis results after Sisagua data selection for the period (2007–2010)**.

Number of records in Sisagua	2007	2008	2009	2010	Total
Reported	34,900	52,561	30,818	50,801	169,080
Reported as not detected	2,727	7,742	5,249	10,183	25,901
Considered as non-valid	9,757	7,324	3,186	5,954	26,221
Considered as valid	25,143	45,237	27,632	44,847	142,859

*See text for clarification of categories*.

### Drinking Water Criteria for the Pesticides with an ADI Established by ANVISA

From the 380 active ingredients approved as pesticides, 210 have ADIs established by ANVISA, and among them 13 are listed in the current drinking water norm (29). For 170 pesticides that do not have established ADIs by ANVISA, 60 of these active ingredients are of biological origin (pheromones, live bait, biological insecticides, plant extracts, among others) (32). Thus, there are 110 active ingredients without an established ADI.

Because of the lack of readily available water quality criteria for several pesticides, these values were calculated for 197 pesticides that are not listed in the current Brazilian drinking water norm. For water quality standards, please see Table S1 in Supplementary Material.

After calculating the drinking water criteria (Table S1 in Supplementary Material) according to the WHO and ANVISA ADIs, we identified some discrepancies in relation to the Brazilian norm standard currently in use. We found, for example, that our calculated value for glyphosate, the most consumed pesticides in Brazil, was 252 mg/L, while the standard established in the current norm is 500 mg/L. For aldicarb, carbofuran, chlorpyrifos, 2,4-D, parathion-methyl, permethrin, and trifluralin, the calculated values are all greater than those in the norm (Table S2 in Supplementary Material). It seems that ADIs different from the ANVISA ones were used in the Brazilian norm^1^ or different allocation factors were applied in the calculations. The values for carbendazim, mancozeb, profenophos, tebuconazole, and terbuphos were identical, indicating that the federal norm applied the same ADI from ANVISA (Table S2 in Supplementary Material). For aldicarb and DDT, DDD and DDE, the criteria suggested by WHO were used. For diuron and mancozeb, the Health Canada ADI was used (15.6 and 30 μg/kg bw, respectively). For the latter, the ADI is the same as the one published by ANVISA. For 2,4-D, alachlor, aldrin/,dieldrin, atrazine, chlordane, endosulfan, endrin, lindane, metolachlor, molinate, pendimenthalin, permethrin, simazine, and trifluralin, the calculation of how the criteria were established was not reported and it appears that the values adopted were from WHO guidelines. For glyphosate, the value used was the same as the previous version of the norm, which was based on a previous WHO report. However, the WHO no longer provides a guideline value for glyphosate using the rationale that this substance would occur in drinking water at concentrations well below those of health concern (10). In this scenario, a new Maximum Allowed Concentration value could be calculated using the ADI set by ANVISA (0.042 μg/kg bw).

## Discussion and Conclusion

A review of the actual exposure of the population to pesticides via drinking water is only possible with a complete and consistent dataset comprising a comprehensive period of study. The Monitoring Information on Water Quality for Human Consumption System (Sisagua) in Brazil is a management tool used by Vigiagua for monitoring the quality of drinking water (40, 41). Therefore, it is of fundamental importance to verify if the analyzed samples are in compliance with the Drinking Water Norm. As described here, several inconsistencies on the monitoring data were identified, and could be attributed to insufficient standardization of the expression of the analytical results, as well as difficulties of the health sector to critically evaluate the data informed by the water suppliers. However, part of this deficiency may also be due to the lack of information about the LOD and LOQ values and the analytical methods used. In 2012, a new Vigiagua form was launched with the requirement to include LOD and LOQ information. Currently, the system is under a redesign process to be adjusted with the new requirements of the MH Ordinance No. 2914/11 (41). This renovated system will be of high importance to the Health sector in the critical evaluation and validation of monitoring data, and will support enforcement actions.

Since the first water quality norm was published in 1977, the number of regulated pesticides has increased (29, 42), reflecting the increasing concern on the use of pesticides in the country. Although the norm lists fewer than 10% of the authorized pesticide active ingredients in Brazil, the current drinking water Ordinance has been assertive on the choice of parameters, including the most widely consumed in the country. It is possible that the established minimum sampling number per year (one sample every six months) is not sufficient considering the consumption and conditions of use of certain pesticides, as well as the differences in each region of the country. The main concern, however, is not on what should or should not be regulated, but whether and how the Ordinance is being enforced. We observed an urgent need for action for the Vigiagua program to work with the health sector to make an effort to have complete pesticides information in the dataset.

Although the Ordinance MH No. 2914/11 included the main active ingredients that have been used in Brazil at the time the norm was issued, important pesticides were left out, such as clomazone, ametryn, tebuthiurom, malathion, picloram, and paraquat dichloride, among others (27, 43, 44). It is important to emphasize that approximately 30% of the 27 pesticides in the current Ordinance are no longer authorized for use in Brazil. Among those that have been canceled are aldrin/dieldrin, chlordane, DDT, endrin, and lindane. Aldicarb, methamidophos, and endosulfan were canceled recently. Most of these substances are persistent organic pollutants (POPs), known as bio magnifier contaminants, and often are monitored and detected in several countries; therefore, they should stay in the norm. However, Sisagua monitoring data suggests that there are some analytical shortcomings in their analyses.

According to Umbuzeiro, the monitoring only of regulated substances usually is not sufficient to ensure the protection of the exposed population (45). There are several other pesticides sometimes used in specific regions that must be analyzed in the drinking water. However, considering the inability to regulate all pesticides with potential occurrence in drinking water, it is necessary that each state or region identify their priority compounds and include them in regional monitoring programs. Another important limitation for the establishment of Brazilian drinking water standards is that several ANVISA monographs, does not inform the ADI values, although in this work we were able to obtain data and offer interim drinking water quality criteria for 197 substances (46). But this approach was not possible for about 110 pesticide active ingredients due to the lack of their ADIs in the ANIVSA monographs.

We also suggest that an allocation factor used for food risk analysis should be used in ANVISA monographs. It would help to determine the proper allocation factor to be used in drinking water criteria as well. This choice is usually guided by physical and chemical properties of the active ingredients. Another important consideration is that, even non-food crop substances should be considered for inclusion in the drinking water norm because they may end up in water bodies too, as verified elsewhere (21, 23, 47–54).

In our study, we observed important differences in ADI reference values between ANVISA monographs and the current Drinking Water Ordinance 2914/11 (e.g., glyphosate, and others; Table S2 in Supplementary Material). Therefore, one intention of the proposed list of drinking water criteria for 197 pesticides is to offer calculated values based on ANVISA’s ADIs to the next revision of the Ordinance. The allocation factor can be discussed and altered if necessary, always in agreement with the food risk assessors, to make sure that no more than 100% of the ADI is used in the water and food reference calculations.

The effective dissemination of water quality information to consumers via Sisagua and by the water suppliers would be also an important form of social control, which could lead to a request to increase the number of monitoring data in Sisagua and to improve the data quality of the system (55). In Europe, for example, there is web-based service called Water Information System for Europe (WISE) provided by a web-portal entry to water related information, with comprehensive information of the quality of inland and marine waters. For users from EU institutions or other environmental administrations, WISE provides input to thematic assessments in the context of EU water related policies; for water professionals and scientists, WISE facilitates access to reference documents and thematic data, which can be downloaded for further analyses; and for the general public, WISE illustrates a wide span of water related information by visualizations on interactive maps, graphs and indicators (56).

There is no doubt that monitoring of pesticides in water is a complex activity which starts with the sampling plan and priority substances that will be analyzed. Chemical analyzes are expensive, require modern equipment and labor skills. As advised by WHO, it is necessary to discuss and assess whether the sampling procedures are appropriately selected, especially sampling sites and sample preservation (10). Therefore, the evaluation and validation of the data needs to occur systematically, with effective actions to improve the information quality. A constant interaction with the water supplier through guidance, reporting and monitoring is also important. In conclusion to our work, we observed that monitoring data of Sisagua during the study period does not assess the exposure of the population to pesticides via the drinking water, especially because of inconsistent and/or absence of data.

The strengths and pitfalls of the Vigiagua program presented in this study represent what was observed during the database evaluation and should not be viewed as a criticism, but as an opportunity for improvement. We believe that the provided information can enhance the awareness and highlight the importance of monitoring toxic chemicals in drinking water as well as in the source waters. The majority of elements highlighted in this study may be relevant in a similar scenario in other developing countries when considering the need to respond to the world’s future drinking water situation. The expectation of this study is to positively mobilize different social actors to the issue, to describe, characterize and identify knowledge gaps and, in particular, to protect the health of people and the planet.

## Conflict of Interest Statement

The authors declare that the research was conducted in the absence of any commercial or financial relationships that could be construed as a potential conflict of interest.

## Footnotes

^1^http://portalsaude.saude.gov.br/images/pdf/2014/julho/24/Documento-Base-de-elaboracao-da-Portaria-MS-2914.pdf
