# Supplementary material for: Pesticides in Drinking Water – The Brazilian Monitoring Program
Source: Front Public Health. 2015 Nov 4;3:246. doi: 10.3389/fpubh.2015.00246 (PMC4631936; doi:10.3389/fpubh.2015.00246)
Supplement: Supplementary file 1 [file table_1.pdf]

PESTICIDES IN DRINKING WATER - THE BRAZILIAN MONITORING PROGRAM

Supplementary material

Table S1. Proposed drinking water quality criteria for 197 pesticides not regulated in Brazil, based on the ADI set by ANVISA (Brazilian Health Surveillance Agency)

Active ingredient	CAS Registry Number	ANVISA code	ADI* (mg/kg body weigth/day)	Drinking Water Criteria (µg/L)
abamectin	71751-41-2	A18	0.002	12
acephato	30560-19-1	A02	0.03	180
acetamiprid	135410-20-7	A29	0.024	140
acibenzolar-S-Methyl	135158-54-2	A38	0.05	300
amicarbazone	129909-90-6	A41	0.02	120
aminopyralid	150114-71-9	A53	0.5	3,000
amitraz	33089-61-1	A23	0.01	60
anilazine	101-05-3	A15	0.1	600
asulam	3337-71-1	A12	0.05	300
aviglycine	49669-74-1	A48	0.002	12
azinsulfuron	120162-55-2	A30	0.1	600
azocyclotin	41083-11-8	A19	0.007	42
azoxystrobin	131860-33-8	A26	0.02	120
benalaxyl	71626-11-4	B38	0.04	240
bentazone	25057-89-0	B03	0.1	600
benthiavalicarb Isopropyl	177406-68-7	B42	0.0099	60
benzyladenine	1214-39-7	B39	0.5	3,000
beta-cyfluthrin	68359-37-5	C61	0.02	120
beta-cypermethrin	65731-84-2	C59	0.01	60

bifenthrin	82657-04-3	B26	0.02	120
bispyribac	125401-75-4	B33	0.01	60
bitertanol	55179-31-2	B24	0.01	60
boscalida	188425-85-6	B41	0.04	240
bromopropilate	18181-80-1	B20	0.03	180
buprofenzin	69327-76-0	B29	0.01	60
cadusafos	95465-99-9	C53	0.0003	1.8
captan	133-06-2	C02	0.1	600
carbaryl	63-25-2	C03	0.003	18
carbosulfan	55285-14-8	C26	0.01	60
carboxin	5234-68-4	C05	0.1	600
carfentrazone-ethyl	128639-02-1	C49	0.03	180
carpropamid	104030-54-8	C62	0.04	240
chinomethionat	2439-01-2	Q01	0.006	36
chlorantraniliprole	500008-45-7	C70	1.58	9,500
chlorfenapyr	122453-73-0	C40	0.03	180
chlormequat	7003-89-6	C15	0.05	300
chlorothalonil	1897-45-6	C18	0.03	180
chromafenozide	143807-66-3	C67	0.09	540
clethodim	99129-21-2	C32	0.01	60
clodinafop	114420-56-3	C68	0.003	18
clofentezine	74115-24-5	C31	0.02	120
clomazone	81777-89-1	C35	0.04	240
cloransulam-methyl	147150-35-4	C50	0.05	300
clothianidin	210880-92-5	C64	0.09	540
cyazofamid	120116-88-3	C66	0.17	1,000
cyfluthrin	68359-37-5	C30	0.02	120
cyhalofop butyl	122008-85-9	C54	0.003	18

cymoxanil	57966-95-7	C09	0.01	60
cypermethrin	52315-07-8	C10	0.05	300
cyproconazole	94361-06-5	C36	0.01	60
cyromazine	66215-27-8	C37	0.02	120
deltamethrin	52918-63-5	D06	0.01	60
diafenthiuron	80060-09-9	D41	0.003	18
diazinon	333-41-5	D10	0.002	12
dicloran	99-30-9	D29	0.01	60
diclosulam	145701-21-9	D43	0.05	300
dicofol	115-32-2	D14	0.002	12
difenoconazole	119446-68-3	D36	0.6	3,600
diflubenzuron	35367-38-5	D17	0.02	120
dimethenamid-P	163515-14-8	D51	0.02	120
dimethoate	60-51-5	D18	0.002	12
dinocap	39300-45-3	D42	0.008	48
diquat	2764-72-9	D21	0.002	12
disulfoton	298-04-4	D23	0.0003	1.8
dithianon	3347-22-6	D24	0.01	60
dodina	2439-10-3	D26	0.01	60
edifenphos	17109-49-8	E01	0.003	18
epoxiconazole	135319-73-2	E22	0.003	18
esfenvalerate	66230-04-4	E18	0.02	120
etefon	16672-87-0	E05	0.05	300
ethionamide	563-12-2	E07	0.002	12
ethiprole	181587-01-9	E29	0.005	30
ethoprophos	13194-48-4	E06	0.0004	2.4
ethoxazole	153233-91-1	E30	0.018	110
ethoxysulfuron	126801-58-9	E23	0.04	240

etofenprox	80844-07-1	E19	0.03	180
famoxadone	131807-57-3	F53	0.006	36
fenamidone	161326-34-7	F55	0.03	180
fenamiphos	22224-92-6	F02	0.0008	4.8
fenarimol	60168-88-9	F03	0.01	60
fenbutatin oxyde	13356-08-6	O09	0.03	180
fenitrothion	122-14-5	F05	0.005	30
fenoterol	25319-90-8	F57	0.008	48
fenoxaprop-P	113158-40-0	F32	0.0025	15
fenpropathrin	39515-41-8	F28	0.03	180
fenpropimorph	67564-91-4	F24	0.003	18
fenpyroximate	134098-61-6	F37	0.01	60
fenthion	55-38-9	F07	0.007	42
fentin	668-34-8	F59	0.0005	3
fipronil	120068-37-3	F43	0.0002	1.2
flazasulfuron	104040-78-0	F48	0.013	78
flonicamid	158062-67-0	F62	0.03	180
fluazifop-p	83066-88-0	F23	0.005	30
flubendiamide	272451-65-7	F66	0.017	100
fludioxonil	131341-86-1	F49	0.04	240
flufenpyr	188490-07-5	F60	0.04	240
flumiclorac-pentyl	87546-18-7	F45	0.3	1,800
flumioxazine	103361-09-7	F46	0.02	120
fluopicolide	239110-15-7	F65	0.08	480
fluquinconazole	136426-54-5	F51	0.05	300
fluridone	59756-60-4	F56	0.08	480
flutriafol	76674-21-0	F36	0.01	60
folpet	133-07-3	F14	0.1	600

fomesafen	72178-02-0	F26	0.003	18
foramsulfuron	173159-57-4	F54	8.5	51,000
formetanate	22259-30-9	F40	0.025	150
gamma-cyhalothrin	76703-62-3	C65	0.001	6
glufosinate	51276-47-2	G05	0.02	120
haloxyfop-p	95977-29-0	H07	0.0003	1.8
hexaconazole	79983-71-4	H09	0.005	30
hexythiazox	78587-05-0	H05	0.03	180
imazalil	35554-44-0	I19	0.03	180
imazapyr	81334-34-1	I12	2.5	15,000
imazaquin	81335-37-7	I08	0.25	1,500
imazethapyr	81335-77-5	I10	0.25	1,500
imidacloprid	138261-41-3	I13	0.05	300
iminoctadine	13516-27-3	I25	0.0006	3.6
indoxacarb	173584-44-6	I21	0.01	60
iodosulfuron-methyl-sodium	144550-36-7	I22	0.03	180
ioxynil octanoate	3861-47-0	O17	0.005	30
iprodione	36734-19-7	I05	0.06	360
iprovalicarb	140923-17-7	I24	0.02	120
isoxaflutole	141112-29-0	I18	0.02	120
kasugamycin	6980-18-3	C07	0.1	600
kresoxim methyl	143390-89-0	C56	0.4	2,400
lambda-cyhalothrin	91465-08-6	C63	0.05	300
lufenuron	103055-07-8	L05	0.02	120
malathion	121-75-5	M01	0.3	1,800
maleic hydrazide	123-33-1	H03	0.3	1,800
mandipropamid	374726-62-2	M45	0.03	180
mesosulfuron methyl-	208465-21-8	M46	1	6,000

mesotrione	104206-82-8	M40	0.005	30
metalaxyl-m	70630-17-0	M31	0.08	480
metamitron	41394-05-2	M33	0.025	150
metconazole	125116-23-6	M34	0.048	290
methidation	950-37-8	M14	0.001	6
methiocarb	2032-65-7	M30	0.02	120
methoxyfenozide	161050-58-4	M32	0.1	600
metiram	9006-42-2	M15	0.03	180
metsulfuron	79510-48-8	M26	0.01	60
mevinphos	26718-65-0	M20	0.0008	4.8
milbemectin	51596-10-2	M38	0.007	42
myclobutanyl	88671-89-0	M27	0.03	180
novaluron	116714-46-6	N09	0.01	60
orthosulfamuron	213464-77-8	O19	0.05	300
paclobutrazol	76738-62-0	P45	0.068	410
paraquat	4685-14-7	P01	0.004	24
penoxsulam	219714-96-2	P51	0.05	300
phorate	298-02-2	F15	0.0005	3
phosmet	732-11-6	F21	0.005	30
picoxystrobin	117428-22-5	P50	0.043	260
pirimicarb	23103-98-2	P10	0.02	120
pirimiphos-methyl	29232-93-7	P12	0.03	180
prochloraz	67747-09-5	P27	0.01	60
procymidone	32809-16-8	P33	0.1	600
prohexadione calcium	127277-53-6	P54	0.2	1,200
propamocarb	24579-73-5	P23	0.1	600
propargite	2312-35-8	P17	0.01	60
propiconazole	60207-90-1	P21	0.04	240

propineb	9016-72-2	P41	0.005	30
prothioconazole	178928-70-6	P53	0.001	6
pymetrozine	123312-89-0	P52	0.0029	17
pyraclostrobin	175013-18-0	P46	0.04	240
pyraflufen	129630-17-7	P49	0.1	600
pyrazophos	13457-18-6	P09	0.004	24
pyrimethanil	53112-28-0	P43	0.2	1,200
pyriproxyfen	95737-68-1	P34	0.1	600
quintozene	82-68-8	Q02	0.01	60
saflufenacil	372137-35-4	S16	0.046	280
spinosad	168316-95-8	E24	0.02	120
spirodiclofen	148477-71-8	E25	0.01	60
spiromesifen	283594-90-1	E26	0.018	110
sulfentrazone	122836-35-5	S09	0.01	60
sulfometuron methyl	74222-97-2	S11	0.02	120
tebufenozide	112410-23-8	T41	0.02	120
tebupirimfos	96182-53-5	T57	0.0002	1.2
teflubenzuron	83121-18-0	T33	0.01	60
tembotrione	335104-84-2	T61	0.0004	2.4
tetraconazol	112281-77-3	T46	0.005	30
thiabendazole	148-79-8	T12	0.1	600
thiacloprid	111988-49-9	T49	0.01	60
thiamethoxam	153719-23-4	T48	0.02	120
thifluzamide	130000-40-7	T52	0.014	84
thiodicarb	59669-26-0	T30	0.03	180
thiophanate-methyl	23564-05-8	T14	0.08	480
thiram	137-26-8	T16	0.01	60
tolyfluanid	731-27-1	T38	0.1	600

triadimefon	43121-43-3	T17	0.03	180
triadimenol	55219-65-3	T31	0.05	300
triazophos	24017-47-8	T18	0.001	6
trifloxystrobin	141517-21-7	T54	0.03	180
trifloxysulfuron	145099-21-4	T55	0.1	600
triflumuron	64628-44-0	T34	0.007	42
triforine	26644-46-2	T25	0.02	120
trinexapac-ethyl	95266-40-3	T56	0.3	1,800
zeta-cypermethrin	52315-07-8	C60	0.005	30
zoxamide	156052-68-5	Z04	0.5	3,000

Note: (*) Values based on ANVISA monographs
