# Supplementary material for: Pesticides in Drinking Water – The Brazilian Monitoring Program
Source: Front Public Health. 2015 Nov 4;3:246. doi: 10.3389/fpubh.2015.00246 (PMC4631936; doi:10.3389/fpubh.2015.00246)
Supplement: Supplementary file 2 [file table_2.pdf]

# PESTICIDES IN DRINKING WATER - THE BRAZILIAN MONITORING PROGRAM

## Supplementary material

**Table S2.** Complete list of pesticides regulated by the Drinking Water Ordinance MH (Ministry of Health) N° 2914/11 and their respective standards compared to the drinking water quality criteria calculated when the ADI (Acceptable Daily Intake) established by ANVISA (Brazilian Health Surveillance Agency) was used

| Active ingredient | CAS Registry Number | Standard from the Water Ordinance 2914/2011 (µg/L) | Calculated Drinking Water Criteria (µg/L) | ADI (ANVISA) mg/kg body weight/day |
|---|---|---|---|---|
| 2,4 D<br>2,4,5 T | 94-75-7<br>93-76-5 | 30 | 60 | 0.01 |
| alachlor | 15972-60-8 | 20 | - | Without ANVISA ADI |
| aldicarb +aldicarbesulfona + aldicarbesulfoxide | 116-06-3<br>1646-88-4<br>1646-87-3 | 10 | 18<br>(Monograph still in use) | Canceled<br>0.003 |
| aldrin<br>dieldrin | 309-00-2<br>60-57-1 | 0.03 | - | Canceled |
| atrazine | 1912-24-9 | 2 | - | Without ANVISA ADI |
| carbendazim+ benomyl | 10605-21-7<br>17804-35-2 | 120 | 120 | 0.02 |
| carbofuran | 1563-66-2 | 7 | 12 | 0.002 |
| chlordane | 5103-74-2 | 0.2 | - | Canceled |
| chlorpyrifos + chlorpyrifos -oxon | 2921-88-2<br>5598-15-2 | 30 | 60 | 0.01 |
| DDT | 50-29-3 | 1 | - | Canceled |

| | | | | |
|---|---|---|---|---|
| DDD<br>DDE | 72-54-8<br>72-55-9 | | | |
| diuron | 330-54-1 | 90 | - | Without ANVISA ADI |
| endosulfan (α, β and salts) | 115-29-7<br>959-98-8<br>33213-65-9<br>1031-07-8 | 20 | - | Canceled |
| endrin | 72-20-8 | 0.6 | - | Canceled |
| glyphosate +<br>AMPA | 1071-83-6<br>1066-51-9 | 500 | 252 | 0.042 |
| lindane (γ HCH) | 58-89-9 | 2 | - | Canceled |
| mancozeb | 8018-01-7 | 180 | 180 | 0.03 |
| methamidophos | 10265-92-6 | 12 | - | Canceled |
| metolachlor | 51218-45-2 | 10 | - | Without ANVISA ADI |
| molinate | 2212-67-1 | 6 | - | Without ANVISA ADI |
| parathion-methyl | 298-00-0 | 9 | 18 | 0.003 |
| pendimethalin | 40487-42-1 | 20 | - | Without ANVISA ADI |
| permethrin | 52645-53-1 | 20 | 300 | 0.05 |
| profenophos | 41198-08-7 | 60 | 60 | 0.01 |
| simazin | 122-34-9 | 2 | - | Without ANVISA ADI |
| tebuconazole | 107534-96-3 | 180 | 180 | 0.03 |
| terbuphos | 13071-79-9 | 1.2 | 1.2 | 0.0002 |
| trifluralin | 1582-09-8 | 20 | 144 | 0.024 |
